# Development of a Thermoelectric and Electromagnetic Hybrid Energy Harvester from Water Flow in an Irrigation System

**DOI:** 10.3390/mi9080395

**Published:** 2018-08-09

**Authors:** Huicong Liu, Jiankang Zhang, Qiongfeng Shi, Tianyiyi He, Tao Chen, Lining Sun, Jan A Dziuban, Chengkuo Lee

**Affiliations:** 1 School of Mechanical and Electric Engineering, Jiangsu Provincial Key Laboratory of Advanced Robotics, Soochow University, Suzhou 215123, China; hcliu078@suda.edu.cn (H.L.); 20165229008@stu.suda.edu.cn (J.Z.); lnsun@hit.edu.cn (L.S.); 2 Department of Electrical & Computer Engineering, National University of Singapore, 4 Engineering Drive 3, Singapore 117576, Singapore; qiongfeng@u.nus.edu (Q.S.); tianyiyi@u.nus.edu (T.H.); 3 Center for Intelligent Sensors and MEMS, National University of Singapore, E6 #05-11F, 5 Engineering Drive 1, Singapore 117608, Singapore; 4 Hybrid-Integrated Flexible (Stretchable) Electronic Systems Program, National University of Singapore, E6 #05-4, 5 Engineering Drive 1, Singapore 117608, Singapore; 5 National University of Singapore Suzhou Research Institute (NUSRI), Suzhou Industrial Park, Suzhou 215123, China; 6 Faculty of Microsystem Electronics and Photonics, Wroclaw University of Science and Technology, 11/17 Janiszewskiego Street, Wroclaw 50-372, Poland; jad@wtm.ite.pwr.wroc.pl

**Keywords:** hybrid energy harvester, water flow, thermoelectric, electromagnetic

## Abstract

A hybrid energy harvester is presented in this paper to harvest energy from water flow motion and temperature difference in an irrigating pipe at the same time. The harvester is based on the integration of thermoelectric and electromagnetic mechanisms. To harvest the water flow motion, a turbine fan with magnets that are attached on the blades is placed inside of the water pipe. Multiple coils turn the water flow energy into electricity with the rotation of the turbine. The thermoelectric generators (TEGs) are attached around the pipe, so as to harvest energy due to temperature difference. For a maximum temperature difference of 55 °C (hot side 80 °C and room temperature 25 °C), twelve serial-connected TEGs can generate voltage up to 0.346 V. Under a load resistance of 20 Ώ, the power output of 1.264 mW can be achieved. For a maximum water flow rate of 49.9 L/min, the electromagnetic generator (EMG) can produce an open circuit voltage of 0.911 V. The EMG can be potentially used as a water flow meter due to the linear relationship between water flow rate and output voltage. Under the joint action of TEG and EMG, the maximum terminal voltage for TEG is 66 mV and for EMG is 241 mV at load resistances of 10 and 100 Ώ, respectively, resulting in a corresponding power output of 0.435 and 0.584 mW.

## 1. Introduction

The irrigation systems have been widely used in agriculture [1]. Instead of irrigating the crops by manpower, various auto-irrigation methods are developed. Sprinkler irrigation is a kind of irrigation mode that spray the water with a certain pressure into the air and then disperse them into small water droplets or mist formation on the plants by water pumps and piping systems [2]. Sprinkler irrigation system can save water without destroying the soil structure and restriction by terrain. In the tropics, the temperature difference between irrigation water and surface temperature can reach to 35–55 °C under sunlight during the dry season. The wasted heat can be utilized to generator power and make the irrigation system more intelligent. At present, various thermoelectric generators (TEGs) have been developed [3,4,5,6,7]. Xiong et al. [8] established a two-stage thermoelectric energy harvesting system to collect the waste heat from the blast furnace slag flushing water. The maximum power output of 0.44 kW is available for an inlet temperature of 100 °C with matched load resistance. He et al. [9] proposed a prototype of thermoelectric module that can generate electricity from the hot water in the pipe heated by solar energy. In irrigation system, water flow motion can also be utilized to generate electricity and power various sensor nodes in agricultural environment. The water flow energy harvesting is based on piezoelectric [10,11,12,13], electromagnetic [14,15,16,17,18], and triboelectric [19,20] approaches. Cho et al. [21] proposed a hydro electromagnetic and piezoelectric energy harvester to power a smart water meter system by transforming the water flow motion in the pipe into electricity, which consists of a turn-buckle type stainless steel waterwheel and two pole magnets. Wang et al. [22] also developed an electromagnetic energy harvester that integrates a Tesla disk turbine, a miniature axial-flux permanent magnet generator, and a ring cover with symmetrical grooves to generate electricity from non-directional water flow in oceans or rivers for remote sensors. In this study, a hybrid energy harvester is investigated to generate energy from the temperature difference and water flow motion in an irrigating pipe, which is based on thermoelectric and electromagnetic mechanisms. To make the irrigation system more intelligent, various sensor nodes or monitors can be added [23,24], e.g., temperature sensor [25] and flow meter [26]. By integrating the TEG and electromagnetic generator (EMG), the self-powered sensor nodes of the irrigation system will be realized in future.

## 2. Design and Fabrication

**Figure 1:**
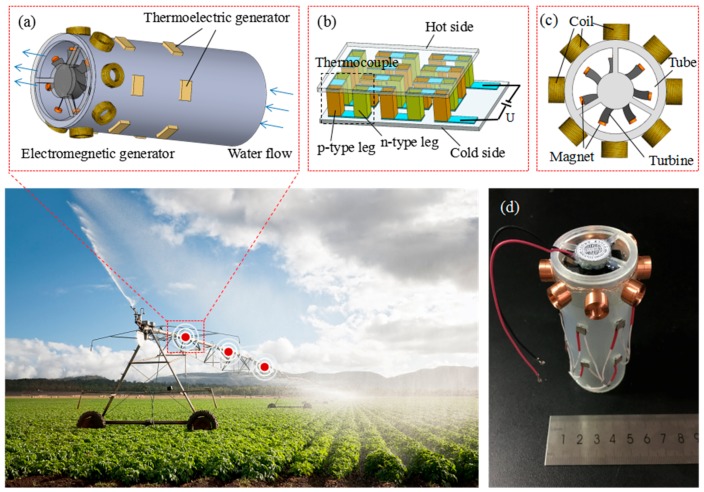
Diagram of hybrid energy harvester in irrigating pipe (**a**) Schematic diagram of hybrid energy harvester; (**b**) Three-dimensional (3-D) structure of thermoelectric generator (TEG); (**c**) Structural diagram of electromagnetic generator (EMG); and, (**d**) Photo of hybrid energy harvester.

In the irrigation system, the diameter of a water pipe usually ranges from 15 to 40 mm. In this work, the inner and outer diameter of the plastic water pipe are designed as 32 and 38 mm, respectively. As shown in Figure 1a, the EMG is mounted at the end of the pipe. The rectangular holes on the side wall of the pipe are designed for TEG assembly. Twelve serial-connected TEGs are divided into two rows and are distributed around the water pipe evenly. The Peltier cooler (TE-8-0.45-1.3 from TE TECHNOLOGY, INC, Traverse City, MI, USA) is used as the TEG. The construction of TEG is shown in Figure 1b. As one side of the device is heated to a temperature greater than the other side, a voltage difference will be built up between the hot side and cold side. By referring to the specification, the effective working area of TEG is about 3.5 mm × 3.5 mm and the size of each thermocouple (TC) is below 1 mm. The components of EMG are coils, magnets, and turbine (Figure 1c). There are eight wound coils that are distributed evenly on the outer side walls of the pipe. The outer and inner diameter of the coil are 9.5 and 5.4 mm, respectively, with 700 turns and wire diameter of 0.13 mm. The resistance of each coil is about 26.8 Ω. The turbine of the cooling fan is used as the turbine of EMG. The frame of the cooling fan will be removed and only the fan part will be used as a turbine. The turbine is fixed to the frame of the water pipe and the magnets are fixed to the tip of turbine blades. The diameter and thickness of the turbine are 28 and 6.9 mm, respectively. When the water flow drives the turbine rotate, the open circuit voltage will be generated in the surrounding coils.

## 3. Working Principle and Simulation

The mechanism of TEG is based on the direct conversion of heat to electrical power through the Seebeck effect [27]. When there is a heat source or a cooling source applied onto each individual side of the thermoelectric materials, electron/hole pairs will be created. A Seebeck voltage potential, which drives the hole/electron flow, is created by the temperature difference between the hot and cold sides of the thermoelectric elements. In a single TC, the net voltage appears between p-type and n-type legs. Since all of the TCs are connected in series, a total net voltage will eventually be produced. Figure 1b shows the three-dimensional (3-D) structure of a TEG device. A number of TCs are connected electrically in series and thermally in parallel. As a temperature gradient is applied to the top and bottom sides, the sum of the Seebeck voltages of the TCs is induced at the generator terminals. The equation of open circuit voltage generated by TEG is as follows, which is proportional to the number of TCs m, the relative Seeback coefficient of the TC materials α, and the temperature difference between cold and hot junctions $\Delta T_{G}$:

(1) $$U_{0}=m\alpha \Delta T_{G}$$

**Figure 2:**
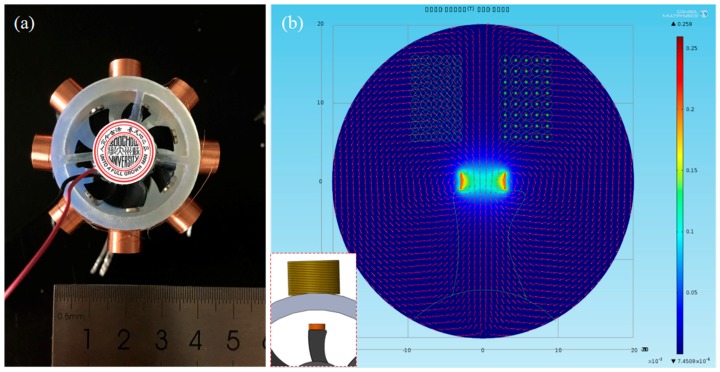
(**a**) Schematic diagram of EMG; and, (**b**) Simulation of the magnetic flux of one magnet across a single coil by COMSOL.

The principle for EMG is based on Faraday’s law of electromagnetic induction [28]. It makes use of the magnetic flux $\Phi_{B}$ through a hypothetical surface Σ whose boundary is a wire loop. Since the wire loop may be moving, Σ is replaced by Σ(t) for the surface. The magnetic flux is defined by a surface integral as:

(2) $$\Phi_{B}=\iint_{\Sigma \left(t\right)}B\left(r,t\right)\cdot dA$$

where dA is an element of surface area of the moving surface Σ(t) and B is the magnetic field (also called magnetic flux density). In more visual terms, the magnetic flux through the coil is proportional to the number of magnetic flux lines. When the magnetic flux changes, Faraday’s law of electromagnetic induction says that the coil acquires an electromotive force (EMF). Equivalently, Faraday’s law states that the EMF is also given by the rate of change of the magnetic flux [29]:

(3) $$E=-N\frac{{d\Phi }_{B}}{dt}$$

where E is the EMF, *N* is the number of turns, and $\Phi_{B}$ is the magnetic flux. The direction of the EMF is given by Lenz’s law. In this paper, when the water in the pipe drive a turbine, the magnet on the tip of turbine blade will rotate, causing the change of magnetic flux across the coil, and the induced current will be produced (Figure 2a). The magnetic flux of one magnet across a single coil is simulated by COMSOL, assuming that the magnet and coil are concentric (Figure 2b). According to the simulation, the EMG can be optimized to increase the output power.

## 4. Experiment and Optimization

**Figure 3:**
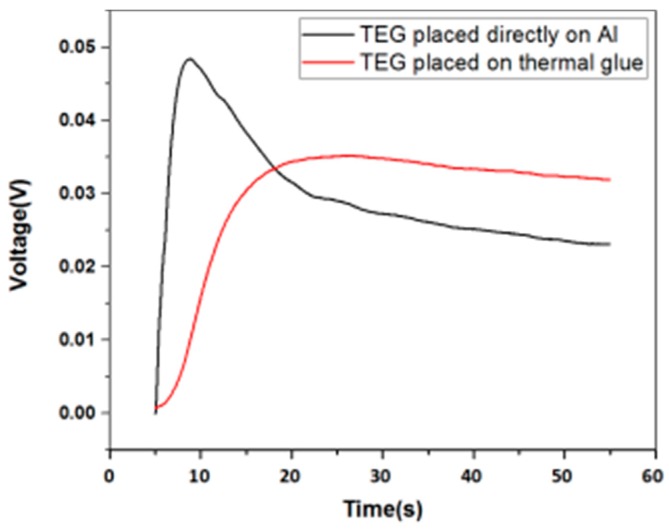
Open circuit voltages of TEG with the hot side placed directly on Al and on thermal glue.

**Figure 4:**
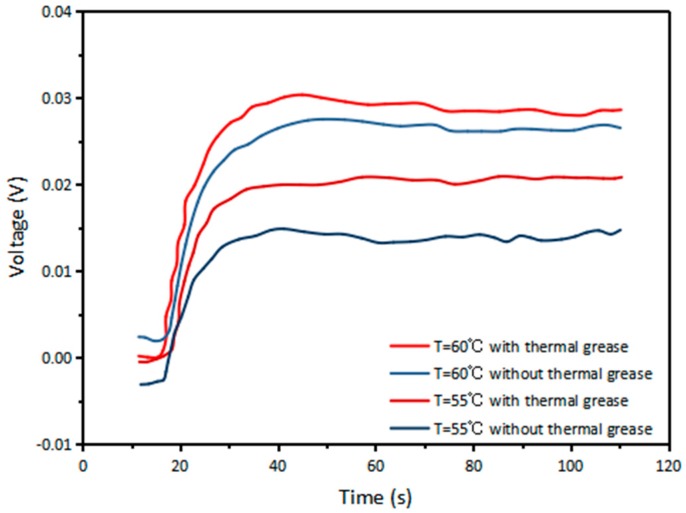
Open circuit voltages of TEG with and without thermal grease attached on the cold side.

There are several aspects to the improvement of TEG: increase the Seebeck coefficient of the material, increase the surface temperature difference, and reduce the thermal conductivity of the material. Figure 3 demonstrates the comparison of the open circuit voltage when the hot side of TEG is placed directly on Aluminum (Al) and on thermal glue. The experiment result shows that at a hot side temperature of 58 °C, the TEG placed on the Al tape directly generates a greater voltage of 46.75 mV, but the voltage drops very quickly. After 50 s, the voltage decreases to 18.25 mV. The generated voltage of TEG placed on the thermal glue is 35 mV, which is lower than the other set. But, the voltage decreases much slower. After 50 s, it still has a value of 32 mV. This is because Al has high thermal conductivity of up to 238 W/(m·K). Using Al tape to seal the holes on the pipe and putting the hot side of TEG directly on the Al tape help the temperature on the hot side increase fast. However, as time goes on, the cold side of TEG also heat up soon, causing the temperature difference to drop very quickly. Although thermal conductive glue has relatively lower thermal conductivity of 2.85 W/(m·K), it can keep the heat for a longer time. As a result, thermal glue is chosen in the subsequent tests. Figure 4 shows that at hot side temperatures of 55 °C and 60 °C (Temperature differences of 23 °C and 28 °C, respectively), TEG with thermal grease attached on the cold side has open circuit voltages of 21 and 31 mV, respectively, while TEG without thermal grease has lower voltages of 15 and 27 mV, respectively. The thermal conductivity of thermal grease is about 4.85 W/(m·K). The aim of applying thermal grease on the cold side of TEG is to increase the speed of heat dissipation on the surface, so that the temperature difference between the cold side and the hot side will not decrease rapidly. Therefore, in the subsequent tests, thermal conductive glue and thermal grease are applied on the hot side and cold side, respectively.

**Figure 5:**
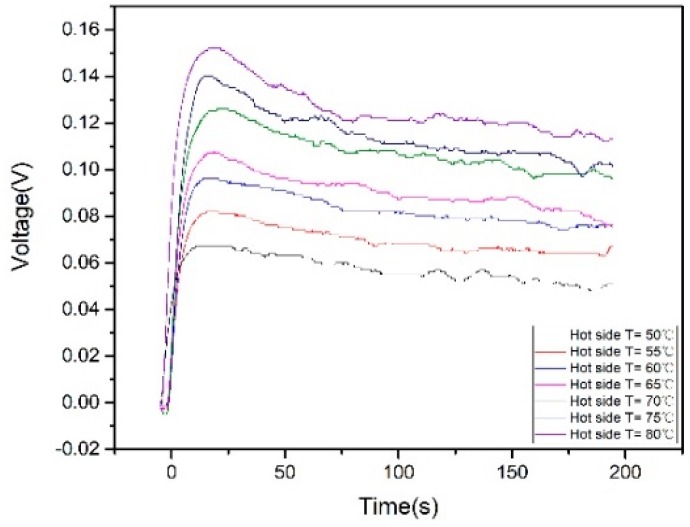
Open circuit voltages of TEGs with different hot side temperatures.

As the TEGs are expected to work for a relative continuous time, the stability of the output voltage of the TEGs over a time period is evaluated. Figure 5 shows the open circuit voltages generated by five TEGs in series with different hot side temperatures. At first, the hot water is poured inside the pipe, TEGs start to generate voltage and reach to a maximum value. Then, the voltage decreases slowly with time. After 200 s, TEGs still have the ability to maintain about 80% of its maximum voltage output (Table 1). The twelve TEG components are connected in series and their terminal voltages and power are tested in terms of different hot side temperatures.

**Figure 6:**
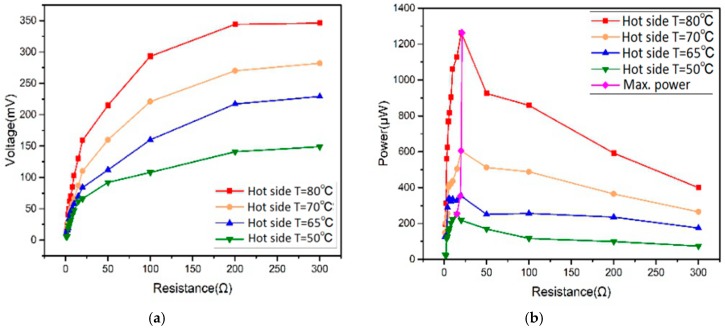
(**a**) Voltage and (**b**) power outputs of TEGs under different load resistances.

Figure 6a is the experimental results of the terminal voltage against different load resistances. The voltage increases rapidly as the load resistance rises, then it undergoes slow increment at higher load resistance. The largest voltages for hot side temperatures of 50 °C, 65 °C, 70 °C, and 80 °C are 149, 229, 282, and 346 mV, respectively. Figure 6b shows the power changes with increasing load resistances. The maximum power outputs for 50 °C, 65 °C, 70 °C, and 80 °C are 0.254, 0.353, 0.605, and 1.264 mW, respectively, under load resistances of 15, 19, 20, and 22 Ω.

**Figure 7:**
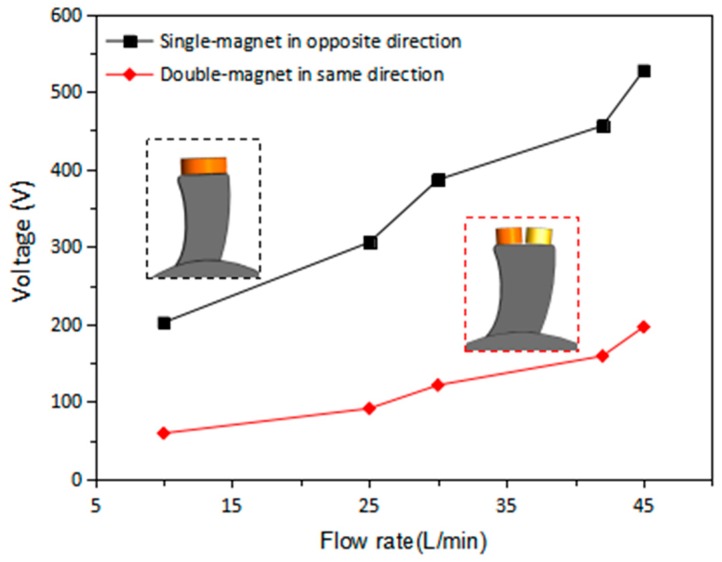
Open circuit voltage of EMG for single-magnet in opposite direction and double-magnet in same direction.

In order to improve the output performance of EMG, the number of magnets on the tip of the turbine blade and the direction of the magnetic pole are taken into consideration. In the single-magnet set, the D3 × 1 mm magnets are placed on the blade in opposite direction. To be specific, the N-pole of a magnet on one blade is placed outward and the N-pole of the magnet on next blade is placed inward. In the double-magnet set, the D2 × 1 mm magnets are placed on the blade in pairs. All of the output voltages of EMG in this paper are peak-to-peak voltages. Figure 7 shows that as the flow rate increases from 10 to 45 L/min, the generated voltage of EMG increases gradually to 527 mV with the magnets being placed in opposite direction, while the maximum voltage of EMG with double-magnet set is only 196 mV. It is found that the voltage output of EMG with single-magnet blade is about twice times higher than that with the double-magnet blade.

**Figure 8:**
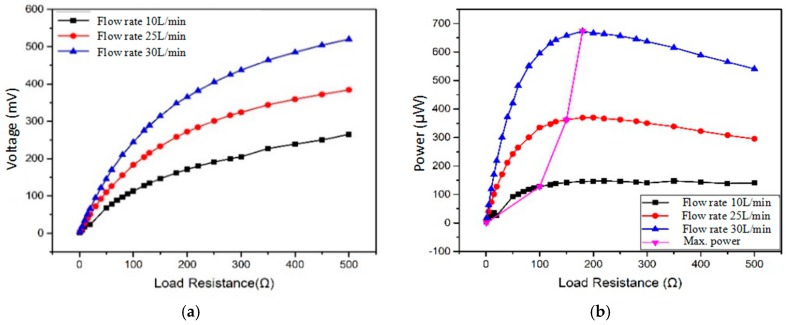
(**a**) Voltage and (**b**) power of EMG with different load resistances.

**Figure 9:**
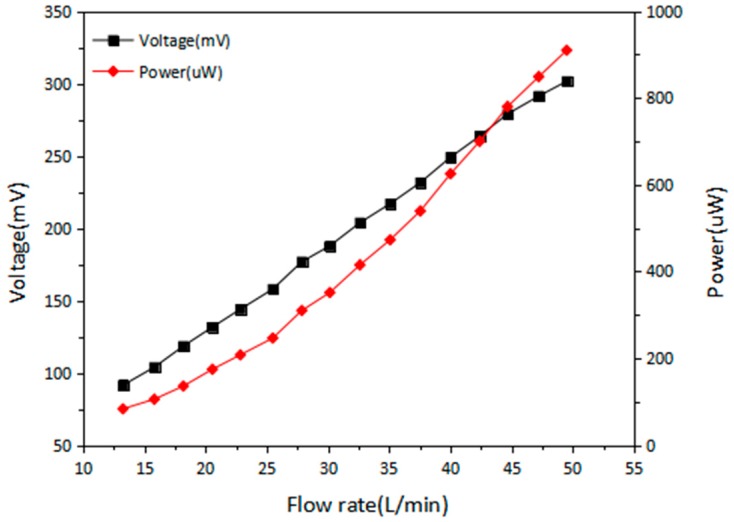
Terminal voltage and power of EMG against water flow rate.

Figure 8 shows the terminal voltage and power outputs of EMG with different load resistances. From Figure 8b, the maximum power of 0.127, 0.362, and 0.673 mW for flow rate of 10, 25, and 30 L/min are achieved at load resistances of 100, 150, and 180 Ω, respectively. Figure 9 shows the voltage and power of EMG versus flow rate under matched load resistance of 100 Ω. At flow rate of 49.5 L/min, the maximum voltage is 302 mV and the corresponding power output is 0.912 mW. It can be observed that there is an approximately linear relationship between the output voltage of EMG and water flow rate. Therefore, it could be a potential application for EMG to be the self-powered water flow meter in irrigation system.

**Figure 10:**
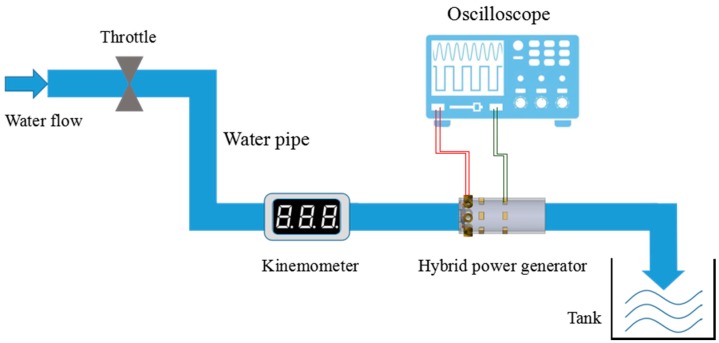
Testing scenario of the collective performance of TEG and EMG.

**Figure 11:**
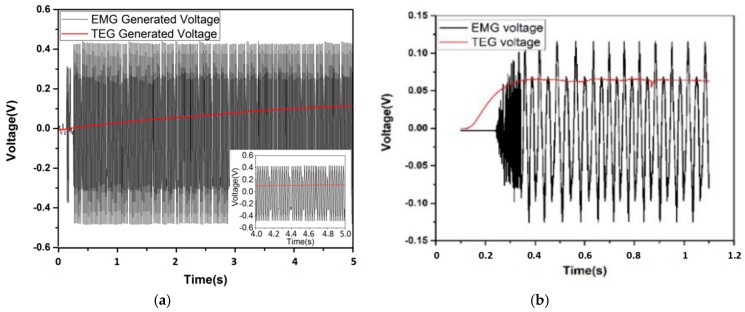
(**a**) Collective open circuit voltage of EMG and TEG; and, (**b**) Collective output voltage of TEG and EMG under load resistance of 10 and 100 Ω.

The integral testing setup is shown in Figure 10, and the output of TEGs and EMG are collected by the oscilloscope. In this scenario, hot water of 60 °C are adopted. Figure 11a shows the voltage waveforms of EMG and TEG. The open circuit voltage of TEGs is 157 mV and EMG is 911 mV. Figure 11b shows a demonstrative graph from oscilloscope when load resistances of 10 and 100 Ω are added to the TEG and EMG circuit, respectively, with a hot side temperature of 60 °C and a flow rate of 38 L/min. The maximum terminal voltages from TEG and EMG are 66 and 241.7 mV, respectively, resulting in power outputs of 0.436 and 0.584 mW.

## 5. Conclusions

This paper presents a hybrid power generator applied in an irrigation system. It integrates the TEGs with water-flow-driven EMG. For the TEG part, it turns out that the TEG performance is improved by putting hot side of TEG on the thermal conductive glue and applying thermal grease on the cold side. For the EMG part, a cooling fan is chosen as the water turbine and the way that placing the single-magnet in opposite direction has a larger output voltage. The result shows that the generated voltage is strongly increased to 527 mV. After the improvement, the TEG and EMG are tested in hot flowing water. For TEG, the experiment shows that the adopted TEG set can have a lasting voltage output over 200 s and maintain about 80% of the maximum output. The largest voltages generated for a twelve TEG set at hot side temperatures of 50 °C, 65 °C, 70 °C, and 80 °C are 149, 229, 282, and 346 mV, respectively, and the power are 0.254, 0.353, 0.605, and 1.264 mW, under load resistances of 15, 19, 20, and 22 Ω. For EMG, the water flow rate and the resulting open circuit voltage follow a linear relationship so that the EMG can be also used as flowmeter. At a flow rate of 49.51 L/min, the maximum voltage is 302 mV and the resulting power output is 0.912 mW with the load resistance of 100 Ω. The collective output is finally tested, with 60 °C flowing hot water at a flow rate of 38 L/min. When load resistances of 10 and 100 Ω are added, the maximum terminal voltage for TEG is 66 mV and for EMG is 241.7 mV, resulting in power outputs of 0.436 and 0.584 mW. Thus, the total output power is better than 1mW, which is sufficient for supporting the power consumption of typical low-power wireless transmission modules that are used in sensor network applications.

## Figures and Tables

**Table 1:** Performance of TEGs with different hot side temperatures.

Hot Side Temperature (°C)	Maximum Open Circuit Voltage (mV)	Voltage after 200 s (mV)	Percentage
80	155	114.025	73.56%
75	147	109.225	74.30%
70	131	102.275	78.07%
65	110	83.15	75.59%
60	97	77.375	79.77%
55	86	69.025	80.20%
50	69	53.75	77.90%
